# Recent advances in managing/understanding the metabolic syndrome

**DOI:** 10.12688/f1000research.17122.1

**Published:** 2019-04-03

**Authors:** Carlos A. Aguilar-Salinas, Tannia Viveros-Ruiz

**Affiliations:** 1 Unidad de Investigación en Enfermedades Metabólicas, Instituto Nacional de Ciencias Médicas y Nutrición Salvador Zubirán, Mexico City, 14008, Mexico; 2 Departamento de Endocrinología y Metabolismo, Instituto Nacional de Ciencias Médicas y Nutrición Salvador Zubirán, Mexico City, 14008, Mexico; 3 Escuela de Medicina y Ciencias de la Salud, Tecnologico de Monterrey, Monterrey, Nuevo Leon, 64710, Mexico; 4 Doctorado de Epidemiología Clínica, Universidad Nacional Autónoma de México, Mexico City, 04510, Mexico

**Keywords:** Metabolic syndrome, lipotoxicity, obesity, type 2 diabetes, hypertriglyceridemia

## Abstract

The metabolic syndrome (MetS) concept gathers in a single entity a set of metabolic abnormalities that have in common a close relationship with ectopic deposit of lipids, insulin resistance, and chronic low-grade inflammation. It is a valuable teaching tool to help health professionals to understand and integrate the consequences of lipotoxicity and the adverse metabolic consequences of insulin resistance. Also, it is useful to identify subjects with a high risk for having incident type 2 diabetes. Systems biology studies have gained a prominent role in understanding the interaction between adipose tissue dysfunction, insulin action, and the MetS traits and co-morbidities (that is, non-alcoholic steatohepatitis, or NASH). This approach may allow the identification of new therapeutic targets (that is, *de novo* lipogenesis inhibitors for NASH). Treatment targets on MetS are the adoption of a healthy lifestyle, weight loss, and the control of the co-morbidities (hyperglycemia, dyslipidemia, arterial hypertension, among others). The long-term goals are the prevention of type 2 diabetes, cardiovascular events, and other MetS-related outcomes. In the last few decades, new drugs derived from the identification of innovative treatment targets have come on the market. These drugs have positive effects on more than one MetS component (that is, hyperglycemia and weight control). New potential treatment targets are under study.

## Introduction

The metabolic syndrome (MetS) concept gathers in a single entity a set of metabolic abnormalities that have in common a close relationship with ectopic deposit of lipids, insulin resistance, and chronic low-grade inflammation. In many cases, a chronic exposure to a positive caloric balance is the driving force for the appearance and progression of this condition. The main traits included in MetS diagnosis are arterial hypertension, central adiposity, hyperglycemia, and atherogenic dyslipidemia ^1^. Several other conditions are related to similar metabolic derangements (that is, liver steatosis, polycystic ovary syndrome, and hyperuricemia) but are not part of the MetS diagnostic criteria. The main long-term complications of MetS are type 2 diabetes ^2^, atherogenesis ^3^, and cognitive impairment ^4^.

Controversy has surrounded the definition of MetS ^5, 6^. Several definitions (composed of dissimilar traits and thresholds) were proposed for its identification over the past 20 years. This clinical construct has been used as a diagnosis, a risk factor, or even an outcome. Contrasting results have been informed when the added value of MetS is compared against the information derived from each one of its individual components for the prediction, prevention, and treatment of its long-term complications. Also, it has been considered a valuable teaching tool to help health professionals to understand and integrate the consequences of a chronic positive caloric balance and the adverse metabolic consequences of excess body weight ^7^.

In this short review, we discuss recent advances in the diagnosis and clinical use of MetS. Also, new findings in its pathophysiology and treatment are highlighted in the final section of the article.

## Metabolic syndrome: definition

MetS definitions are composed of one or more clinical variables or biomarkers of the presence of the main traits ( Table 1) included in the MetS concept (central adiposity, hypertension, high triglycerides, low high‐density lipoprotein cholesterol, and hyperglycemia) ^8– 13^. The selection of the variables and its thresholds has been a complex task because the main MetS traits could be assessed by several indicators that provide complementary information (for example, fasting plasma glucose, HbA1c, or even the 2-hour glucose concentration after an oral glucose load for the “hyperglycemia” trait). In addition, authors have applied diverging conceptual models to build their diagnostic frame. For example, the International Diabetes Federation and the National Cholesterol Education Program (NCEP) definitions are focused on central obesity; in contrast, the World Health Organization definition is focused on insulin resistance. As a result, these tools identify heterogeneous populations as having the same condition. The existence of several MetS definitions created inconsistent results and confusion. However, the practicality of the NCEP criteria ^9^ (or its harmonized version ^8^) has made this diagnostic tool the most accepted diagnostic criterion. Its use has been included in some obesity and cardiovascular risk guidelines ^14^. However, it is questionable that the inclusion of MetS in the clinical assessment or the prognostic evaluation of at-risk cases for having non-communicable diseases provides more information than that obtained by the evaluation of each one of the MetS traits. As a consequence, several groups have proposed alternative approaches intended to be used in research. To avoid arbitrary decisions in the selection of the thresholds to define abnormal values of the MetS traits, our group proposed a score based on the population-based distribution of each one of the MetS components ^15^. One point was given per decile for every component. The total number of points accumulated was used to classify subjects. Individuals in the upper quartile of the point scale (≥39 points) had an odds ratio to have incident diabetes significantly greater than that found with the NCEP criteria (12.71 versus 11.14). The advantage of this approach is the detection of subjects at the extreme of the range of diabetes risk and the ability to estimate the risk as a continuum. A similar approach was applied to develop the MetS-Z score, which is based on the weighted contribution of each component on a sex- and race-specific basis derived from the US National Health and Nutrition Examination Survey. This approach is capable of predicting incident type 2 diabetes and cardiovascular events ^16^. In a recent report, MetS-Z score was applied to assess the response to a healthy lifestyle program in the Diabetes Prevention Program. The 1- to 5-year risk of incident diabetes was strongly associated with 1-year changes in MetS-Z score and waist circumference (hazard ratios for a 1–standard deviation increase = 1.80), whereas the risk of cardiovascular disease was associated with a 1-year change in MetS-Z score, glucose, and systolic blood pressure ^17^. These observations suggest that the MetS definition is still a work in progress. The use of continuous scores may be an option to improve the identification of at-risk individuals and to assess their response to therapy. Some of the continuous definitions are highlighted in Table 2 ^17– 21^. However, its translation into clinical practice will require the demonstration of a clear advantage of its use over current conventional practice. As a consequence, MetS cannot be considered a treatment goal or an outcome ^22^.

**Table 1.** Diagnostic criteria for metabolic syndrome.

	WHO 1998	EGSIR 1999	ATP III 2001	AACE 2003	IDF 2005	AHA/NHLBI 2005	AHA/NHLBI+ IDF 2009
Definition of MetS	Insulin resistance + any other two components	Plasma insulin concentration >75th percentile of non- diabetic patients + any of two components	Any of three out five components	Insulin resistance + any other component	Obesity + at least two components	At least three components	Any three components
Components of MetS							
Obesity	Waist/hip ratio >0.9 in males and >0.85 in females or BMI >30 kg/m ^2^	Waist circumference ≥94 cm in males and ≥80 cm in females	Waist circumference >102 cm in males and >80 cm in females	BMI >25 kg/m ^2^	BMI >30 kg/m ^2^ or specific gender and ethnicity waist circumference cutoffs	Waist circumference >40 inches in males and >35 inches in females	Raised waist circumference (population- and country-specific definitions)
Elevated triglycerides	TG ≥150 mg/dL	TG ≥150 mg/dL or treatment of this lipid abnormality	TG ≥150 mg/dL or treatment of this lipid abnormality	TG ≥150 mg/dL	TG ≥150 mg/dL or treatment of this lipid abnormality	TG ≥150 mg/dL or treatment of this lipid abnormality	TG ≥150 mg/dL or treatment of this lipid abnormality
Decreased HDL	HDL <35 mg/dL: males <39 mg/dL: females	HDL <39 mg/dL: males and females or treatment of this lipid abnormality	HDL <40 mg/dL: males <50 mg/dL: females Or specific treatment for this lipid abnormality	HDL <40 mg/dL: males <50 mg/dL: females	HDL <40 mg/dL: males <50 mg/dL: females Or specific treatment for this lipid abnormality	HDL <40 mg/dL: males <50 mg/dL: females Or specific treatment for this lipid abnormality	HDL <40 mg/dL: males <50 mg/dL: females Or specific treatment for this lipid abnormality
Hypertension	BP ≥140/90 mm Hg	BP ≥140/90 mm Hg or antihypertensive medication	SBP ≥130 or DBP ≥85 mm Hg or taking medication for hypertension	BP ≥130/85 mm Hg	SBP ≥130 or DBP ≥85 mm Hg or treatment of previously diagnosed hypertension	BP ≥130/85 mm Hg or taking medication for hypertension	BP ≥130/85 mm Hg or taking medication for hypertension
Hyperglycemia	Impaired glucose tolerance, impaired fasting glucose, or lowered insulin sensitivity	Fasting plasma glucose >110 mg/dL	Fasting plasma glucose >110 mg/dL (modified in 2004) or taking medicine for high glucose	Impaired glucose tolerance or impaired fasting glucose (but not diabetes)	Fasting plasma glucose >100 mg/dL or previously diagnosed type 2 diabetes	Fasting plasma glucose >100 mg/dL or taking medicine for high glucose	Fasting plasma glucose >100 mg/dL or taking medicine for high glucose
Other	Urine albumin ≥20 µg/min or albumin: creatinine ratio ≥30 mg/g	None	None	Other features of insulin resistance (family history of diabetes, polycystic ovary syndrome, sedentary lifestyle)	None	None	None

AACE, American Association of Clinical Endocrinologists; AHA/NHLBI, American Heart Association/National Heart, Lung, and Blood Institute; ATP, Adult Treatment Panel; BMI, body mass index; BP, blood pressure; DBP, diastolic blood pressure; EGSIR, European Group for the Study of Insulin Resistance; HDL, high‐density lipoprotein; IDF, International Diabetes Federation; MetS, metabolic syndrome; SBP, systolic blood pressure; TG, triglycerides; WHO, World Health Organization

**Table 2.** Some continuous scores for the diagnosis of metabolic syndrome.

Authors	Statistical approach	Clinical use	Reference
Aguilar-Salinas *et al*.	Population-based distribution of the main traits of metabolic syndrome	Prediction of incident diabetes	15
DeBoer *et al*.	Metabolic syndrome–Z score	Prediction of incident diabetes and cardiovascular events Assessment of response to a diabetes prevention program	16 17
Magnussen *et al*.	Principal component analyses Sum of standardized Z scores Sum of standardized residuals	Prediction of cardiometabolic outcomes	18
Wijndaele *et al*.	Principal component analyses	Assessment of the severity of the metabolic abnormalities	19
Janghorbani and Amini	Age and gender Z scores	Prediction of incident diabetes	20
Kang *et al*.	Sum of points derived from a Cox regression model	Prediction of cardiovascular outcomes	21

Nearly 30% of obese individuals are free of metabolic co-morbidities ^23^. This phenotype has been called “metabolically healthy obesity” (MHO). The MetS definitions have been adapted to identify this condition. However, more than five versions derived from the NCEP definition have been proposed. We should learn from the past ^24^. The definition of a clinical construct should be based on evidence and a conceptual model. The MHO is a biological model with limited clinical implications.

## Pathogenesis of metabolic syndrome

The underlying mechanism by which the MetS components share aspects of their pathophysiology is a matter of intense research. The capacity of the adipose tissue to expand and store energy substrates plays a critical role in its pathophysiology. However, other metabolic pathways should be involved since the same metabolic abnormalities seen in MetS could happen in lean individuals ^25^. Systems biology approaches and omics-based research have been critical to develop new holistic models ^26^. This is the case for the recently published systems biology studies carried out in patients with non-alcoholic steatohepatitis ^27^. Based on this approach, the involvement of several metabolic pathways ( *de novo* lipogenesis, beta oxidation, pyruvate utilization, and serine and glutathione synthesis) was recognized. Some of them are activated to handle the excessive flux of energy precursors found in MetS and to prevent the accumulation of oxygen radicals. Furthermore, the critical role of pyruvate kinase liver red cell ( *PKLR*) as a determinant of triglyceride accumulation in the liver was identified ^28^. In summary, lipotoxicity, low-grade chronic inflammation, and insulin resistance are the prevailing complementary mechanisms that the majority of groups consider the core of MetS pathogenesis. Its description goes beyond the scope of this review. Interested readers will find the description of the state of the art of this field in outstanding reviews included in the References section ^29– 33^.

In the near future, the study of the contribution of new approaches (that is, metagenomics, impact of environmental factors on the epigenome, and expansion or differentiation of adipose tissue, among others) may become novel evidence to improve innovative targets for prevention or treatment ^34– 37^.

## Management of metabolic syndrome

**Figure 1.**
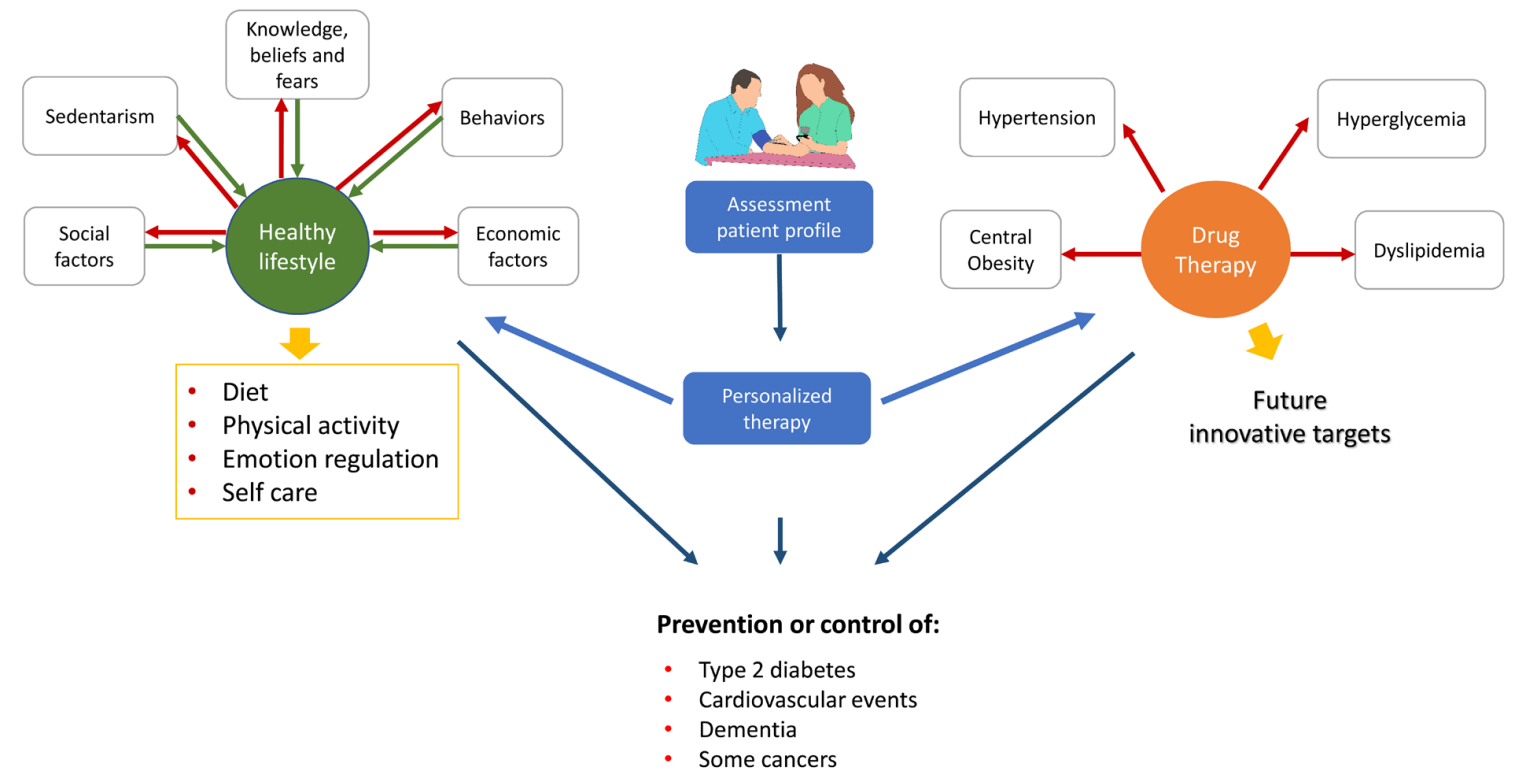
Treatment of metabolic syndrome. A personalized approach should be applied in the adoption of a healthy lifestyle and in the prescription of drug therapy. Adherence is a major challenge that could be overcome by identifying the patient profile, estimating the empowerment and addressing the main or most common barriers to include therapy in the daily routines.

Treatment targets on MetS are the adoption of a healthy lifestyle, weight loss, and the control of co-morbidities (hyperglycemia, dyslipidemia, and arterial hypertension, among others) ^38^ ( Figure 1). The long-term goals are the prevention of type 2 diabetes, cardiovascular events, and other MetS-related outcomes (that is, dementia and chronic liver disease).

The adoption of a healthy lifestyle is the cornerstone of MetS treatment. Diet, physical activity, sleep, emotion control, peer support, and avoidance of tobacco, alcohol, and other drugs/medications that alter satiety or body weight are key targets of any healthy lifestyle program. Each one requires a systematic assessment and a patient-centered intervention plan. Knowledge, beliefs, fears, barriers to achieve adherence to therapy, and motivation to change should be evaluated for each target ^39^. Algorithms or intervention plans based on patient profiles are conventional strategies to intervene. In many health systems, innovative approaches (that is, app-based support systems and web-based interventions) have reinforced the intervention plans. However, many challenges remain unsolved. In a large proportion of cases, the effect of the intervention is short-lived. The intervention does not alter major environmental and sociodemographic factors that determine health-related conduct; as a result, unhealthy behaviors persist.

The MetS concept could be used as a teaching tool in healthy lifestyle programs. This approach was applied in the The Reversal Intervention for Metabolic Syndrome (TRIMS) study ^40^. During a 55-minute session, participants discussed with health professionals the contributing factors for having MetS and the health risks that could be avoided. However, the impact of the session was not analyzed separately from the overall intervention. The same limitation is found in meta-analyses that have measured the effect of lifestyle programs on the prevention of type 2 diabetes and other MetS long-term risks ^41^.

A prime objective is weight loss. Even a small weight loss (3% or more) results in an improvement in several MetS components. The most likely explanation for that is the high turnover rate of the intra-abdominal fat depots; as a result, a slight weight loss reduces the liver exposure to fatty acids and other pro-inflammatory mediators. A sustained weight loss greater than 10% could be enough to reverse glucose intolerance, arterial hypertension, and several of the lipoprotein abnormalities. The best source of evidence of the prominent role of weight loss in MetS treatment is bariatric surgery, in which a 30 to 40% weight loss is capable of reversing the majority of the MetS abnormalities ^42^. This action is not exclusive to bariatric surgery ^43^. In addition, complementary mechanisms of bariatric surgery beyond weight loss (that is, farnesoid X receptor [FXR] activation, changes in incretin secretion, or modification of microbiota) have been postulated ^44^. Despite the above, weight loss is an unmet need for many MetS cases. Few cases attain ideal body weight or maintain weight loss over time.

The third treatment target is the control of co-morbidities. Effective drug therapies are available for treating hyperglycemia, arterial hypertension, and dyslipidemia. As a result, the accomplishment of this target is the most likely approach to be implemented in primary care centers to prevent MetS-related outcomes. Metformin, statins, angiotensin-converting enzyme (ACE) inhibitors, and angiotensin receptor blockers are among the most frequent drugs used to treat co-morbidities. In the last few decades, new drugs derived from the identification of innovative treatment targets have come on the market. This is the case with the glucagon-like-peptide 1 (GLP-1) agonists, sodium-glucose co-transporter 2 (SGLT2) inhibitors, and dipeptidyl peptidase 4 (DPP4) inhibitors ^44^. These drugs have positive effects on more than one MetS component (that is, hyperglycemia and weight control). Preliminary data suggest that GLP-1 agonists and SGLT2 inhibitors may reduce cardiovascular or renal outcomes ^45, 46^. Long-term studies are under development to demonstrate the effectiveness of such interventions. Cost-effectiveness analyses are needed to integrate these therapies in the majority of health systems. New potential treatment targets are under study. This is the case with FXR agonists ^47^, new peroxisome proliferator-activated receptor (PPAR)-gamma or PPAR-alpha modulators ^48, 49^, GIP/GLP1 dual agonists ^50^, dual SGLT1/SGLT2 inhibitors ^51^, G protein–coupled receptor (GPCR/GPR) agonists ^52^, apical sodium-dependent bile acid transporter (ASBT) inhibitors ^53^, chemokine receptor 2 and 5 antagonists, fibroblast growth factor 19 agonists ^54^, and modulators of microbiota or their products among many others MetS treatment is an area under intense investigation. However, owing to long-term safety issues, the introduction of new agents is not as fast as needed.

## Conclusions

MetS is a topic in which many disciplines are interested. Besides which definition is used, the MetS concept is a valuable tool in medical education. It provides an overview of the complex mechanisms by which chronic exposure to a positive caloric balance or lipotoxicity (or both) causes long-term complications. Implementing cost-effective therapies and developing new therapeutic strategies for MetS should be considered high-priority areas.
